# A prospective multi-center registry concerning the clinical performance of laparoscopic colorectal surgery using an absorbable adhesion barrier (INTERCEED^®^) made of oxidized regenerated cellulose

**DOI:** 10.1007/s00595-019-01816-7

**Published:** 2019-04-29

**Authors:** Jun Watanabe, Fumio Ishida, Hideyuki Ishida, Yosuke Fukunaga, Kazuteru Watanabe, Masanori Naito, Masahiko Watanabe

**Affiliations:** 1 Department of Gastroenterological Surgery, Yokohama City University Graduate School of Medicine, 3-9, Fukuura, Kanazawa-ku, Yokohama, 236-0004 Japan; 2 Digestive Disease Center, Showa University Northern Yokohama Hospital, Yokohama, Japan; 3 Department of Digestive Tract and General Surgery, Saitama Medical Center, Saitama Medical University, Saitama, Japan; 4 Department of Gastroenterological Surgery, Cancer Institute Hospital, Japanese Foundation for Cancer Research, Tokyo, Japan; 5 Department of Surgery, NTT Medical Center Tokyo, Tokyo, Japan; 6 Department of Colorectal Surgery, Kitasato University School of Medicine, Sagamihara, Japan

**Keywords:** INTERCEED^®^, Oxidized regenerated cellulose, Colorectal surgery, Small bowel obstruction, Adhesion barrier

## Abstract

**Purpose:**

The aim of this prospective multi-center registry was to evaluate the safety and clinical performance of INTERCEED^®^ in laparoscopic colorectal surgery.

**Methods:**

This study was a prospective, multi-center, single-arm registry wherein patients who received INTERCEED^®^ in laparoscopic colorectal surgery were registered consecutively (UMIN-CTR 00001872). The primary outcome was the incidence rate of postoperative adhesive small intestinal obstruction within 6 months. The secondary outcomes were reoperation related to postoperative bleeding and anastomotic leak, surgical site infection (SSI) and anastomotic leak.

**Results:**

Between March 2012 and March 2015, a total of 202 patients were enrolled from six institutions. INTERCEED^®^ was not applied in two patients, so 200 patients were analyzed using the full analysis set population. The incidence rate of postoperative adhesive intestinal obstruction was 1.0% (2/200). The total SSI rate was 3.5% (7/200), the deep incisional SSI rate was 0.0% (0/200), and the organ SSI rate was 0.0% (0/200). The incidence of anastomotic leak was 1.0% (2/200). Reoperation was performed in two cases: one for anastomotic leak and the other as cardiac surgery due to heart disease.

**Conclusions:**

Using INTERCEED^®^ in laparoscopic colorectal surgery is safe and may be useful for preventing postoperative adhesive small intestinal obstruction.

## Introduction

Postoperative adhesion formation is the most common complication of abdominal or pelvic surgeries, affecting up to 93% of patients, although the literature notes a lack of awareness in the surgical community [1]. Certain surgical procedures are considered to carry a high risk for adhesion formation, including ovarian surgery, endometriosis surgery, tubal surgery, myomectomy and, ironically, adhesiolysis [2]. A recent systematic review was performed by Okabayashi et al. to estimate the incidence of postoperative adhesion at a second-look operation in patients undergoing abdominal surgery [3]. They found that the weighted mean rate of adhesion for all patients was 54%. A subgroup analysis by surgical specialty showed weighted mean rates of 66% after gastrointestinal surgery, 51% after gynecologic surgery and 22% after urologic surgery. An additional analysis by surgical procedure type revealed weighted mean rates of 61% for cholecystectomy, 67% for total colectomy, 41% for cesarean section and 64% for myomectomy [3].

A 2014 systematic review and meta-analysis reported on the use of four adhesion prevention adjuvants (oxidized regenerated cellulose, hyaluronate carboxymethylcellulose, icodextrin liquid or polyethylene glycol gels) compared to no treatment in abdominal surgery [4]. Twenty-eight trials assessing 5191 patients were included in the meta-analysis (11 oxidized regenerated cellulose,9 hyaluronate carboxymethylcellulose, 4 icodextrin liquid and 4 polyethylene glycol gels). Both oxidized regenerated cellulose and hyaluronate carboxymethyl cellulose significantly reduced the incidence of site-specific adhesion formation. Oxidized regenerated cellulose reduced the incidence of adhesion in gynecological surgery. However, the rate of serious adverse events was not investigated for any of the four agents.

INTERCEED^®^ (Johnson & Johnson, New Brunswick, NJ, USA) is an absorbable adhesion barrier composed of 100% oxidized regenerated cellulose polysaccharide consisting of residues of glucuronic acid and glucose, with β linkages. Macrophages/mononuclear phagocytes contain lysosomal enzymes (beta-glucuronidase and beta-glucosidase), which are capable of degrading these β linkages. INTERCEED^®^ is used as an adjuvant in general abdominal and gynecological operations, where it is surgically implanted after meticulous hemostasis has been achieved to reduce the occurrence of postoperative adhesion.

The clinical use of INTERCEED^®^ has been reported in various gynecological operations for the prevention of adhesion of the peritoneal membrane and the intestinal tract [5–14]. In gastrointestinal surgery, hyaluronate carboxymethylcellulose has been used for adhesion prevention [15–20]. However, hyaluronate carboxymethylcellulose is not suited from the perspective of operability in cases of trocar-assisted laparoscopic surgery or for use in narrow spaces [21]. A previous report on the use of INTERCEED^®^ for laparoscopic colorectal surgery was only a single-center, randomized controlled trial of a small number of cases [21].

The aim of this prospective, multi-center registry was to evaluate the safety and clinical performance of the synthetic absorbable adhesive barrier INTERCEED^®^ in laparoscopic colorectal surgery.

## Materials and methods

### Patients

This study was a prospective, multi-center, single-arm registry wherein laparoscopic colorectal surgery patients who received INTERCEED^®^ were registered consecutively at six institutions in Japan. The study protocol was approved by the ethics advisory committee and the institutional review board of each participating hospital before the study was initiated. The study was registered in the Japanese UMIN Clinical Trials Registry as UMIN000018727 [https://www.umin.ac.jp/ctr/index.htm], and all patients provided their written informed consent before registering in the study. Patients who were ≥ 20 years of age and undergoing laparoscopic colorectal surgery were eligible for this study. The inclusion/exclusion criteria for this study are shown in Table 1.

**Table 1:** The inclusion/exclusion criteria for this study

[Inclusion criteria]
Subjects who are ≥ 20 years of age
Subjects undergoing abdominal laparoscopic surgery
Subjects willing to participate in the study and who have provided their written informed consent
Subjects in whom the use of INTERCEED^®^ may be considered during the planned surgery
[Exclusion criteria]
Subjects requiring conversion from a laparoscopic procedure to open surgery
Patients in whom significant adhesive disease is already present at the inception of the procedure, requiring adhesiolysis, which affects the surgical time
Subjects in whom complete excision of the tumor was not achieved
Subjects with intraoperative intraperitoneal administration of chemotherapy
Subjects with severe hepatic dysfunction, renal failure, heart disease and infectious disease
Subjects in whom alternate adhesive prevention methods are used
Subjects in whom complete hemostasis could not be achieved at the location where INTERCEED^®^ was going to be used
Subjects in whom the location where INTERCEED^®^ was going to be used is considered infected
Subjects with a history of severe drug allergy
Subjects with an allergy to oxidized regenerated cellulose
Subjects in whom the treating physician does not feel that the application of INTERCEED^®^ is appropriate

### Procedure

At the final step of the surgery, complete hemostasis was achieved before the use of INTERCEED^®^. Liquids were completely removed from within the abdomen, and INTERCEED^®^ was cut to an appropriate size (approximately 3–5 mm larger than the site requiring adhesion prevention, such as an umbilical small incision and exfoliated surface). INTERCEED^®^ was placed over or between the exposed surfaces to prevent the exposed surface from adhering to the adjacent tissue. The INTERCEED^®^ was inserted via the umbilical small incisional when applied under an umbilical small incision and via the trocar when applied to the pelvic floor. Care was taken not to wrap the anastomotic site with INTERCEED^®^. Securing with sutures was deemed unnecessary. Immediately before abdominal closure, a single layer of INTERCEED^®^ was applied under dry conditions. If the INTERCEED^®^ sheet was stained black, sufficient prevention of adhesion could not be expected, so the sheet was promptly removed, and a new one was applied after hemostasis had been achieved again. If a single sheet was insufficient to cover the entire target site, additional sheets were used, with overlap by 3–5 mm to ensure complete coverage of the target site. The sheets were moisturized with up to 2 mL of physiological saline per 3 × 4 in. (7.6 × 10.2 cm) to ensure complete attachment to the tissue.

### Endpoints

The primary outcome of this study was the incidence rate of postoperative adhesive small intestinal obstruction within 6 months. Adhesive small intestinal obstruction was defined as rehospitalization or prolonged hospitalization in a patient presenting with clinical symptoms of adhesive small intestinal obstruction requiring long tubing, along with liquid-level findings accompanied by small intestinal dilatation on an X-ray examination. Whether or not prolonged hospitalization was required was determined by each attending physician.

The secondary outcomes were reoperation related to postoperative bleeding and anastomotic leak, surgical site infection (SSI) and anastomotic leak.

### Statistical analyses

The sample size in this study was estimated based on the incidence of postoperative adhesive small intestinal obstruction. By referring to the data in the published literature, the incidence of postoperative adhesive small intestinal obstruction in this study was assumed to be 1.5%. The sample size required to allow the detection of at least one patient with postoperative adhesive small intestinal obstruction in this study at a probability of ≥ 95% was calculated to be ≥ 198. Based on this result, the target sample size in this study was set at 200.

In this study, the data analyses of the primary and secondary outcomes were performed in the full analysis set (FAS) population, consisting of all registered patients.

Continuous values were presented using the number of subjects, mean, standard deviation, minimum and maximum. Binominal values were presented as the frequency and percentage. Clinical data including the primary outcome items were presented as the frequency, percentage and 95% confidence interval.

## Results

**Fig. 1:**
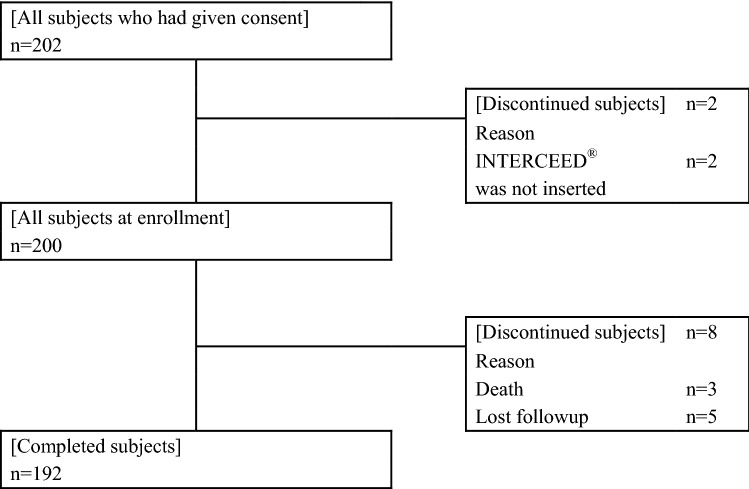
Outline of the patient selection process in the present study

Between March 1, 2012, and March 31, 2015, a total of 202 patients were collected from six institutions. INTERCEED^®^ was not inserted in two patients, so 200 patients were analyzed using the FAS population (Fig. 1). The clinical characteristics of the 200 patients are presented in Table 2. The surgical procedure and outcomes are summarized in Table 3. The site of INTERCEED^®^ application was on the omentum under an umbilical incision (71.0%), on the non-omentum under an umbilical incision (32.0%) and exfoliation on the pelvic floor (1.5%).

**Table 2:** The clinical characteristics of the 200 patients

		Mean ± SD (minimum, median, maximum)
		% (n/N)
Number of subjects	*N*	200
Age, years	Mean ± SD	68.6 ± 10.8 (29, 69.0, 92)
Gender	% (n/N)	
Male		52.5% (105/200)
Female		47.5% (95/200)
Weight (kg)	Mean ± SD	57.92 ± 12.58 (34.0, 55.75, 105.3)
Height (cm)	Mean ± SD	159.97 ± 9.28 (140.3, 160.00, 183.0)
BMI, kg/m^2^	Mean ± SD	22.46 ± 3.43 (14.8, 22.25, 34.2)
Diagnosis	% (n/N)	
Cecal cancer		5.5% (11/200)
Ascending colon cancer		13.5% (27/200)
Transverse colon cancer		11.0% (22/200)
Descending colon cancer		5.5% (11/200)
Sigmoid colon cancer		27.0% (54/200)
Rectosigmoid cancer		10.5% (21/200)
Rectum cancer		21.5% (43/200)
Crohn’s disease		0.0% (0/200)
Ulcerative colitis		0.0% (0/200)
Other		7.0% (14/200)
Stage grouping	% (n/N)	
0		2.5% (5/200)
I		33.5% (67/200)
II		32.5% (65/200)
III		22.0% (44/200)
IV		4.0% (8/200)
Combination therapy, ≤ 90 days	% (n/N)	
Chemotherapy		5.0% (10/200)
Radiotherapy		1.0% (2/200)
Immunotherapy		0.0% (0/200)
Long term steroid medication	% (n/N)	1.0% (2/200)
Complication/ history	% (n/N)	
Diabetes mellitus		21.5% (43/200)
Hypertension		35.5% (71/200)
Encephalopathy		4.5% (9/200)
Angina pectoris, ≤ 30 days		2.0% (4/200)
Myocardial infarction, ≤ 6 months		0.0% (0/200)
Arterial occlusive disease		0.5% (1/200)
Congestive heart failure, ≤ 30 days		1.5% (3/200)
Pneumonia at OR admission		0.0% (0/200)
COPD		4.5% (9/200)
Acute renal failure, ≤ 24 h		0.0% (0/200)
Dialysis, ≤ 14 days		0.5% (1/200)
Weight loss ≥ 10%, ≤ 6 months		2.0% (4/200)
Abdominal dropsy		0.0% (0/200)
Blood coagulation disorder		1.5% (3/200)
Smoking		9.5% (19/200)
History of abdominal open surgery		30.0% (60/200)

*BMI* body mass index, *OR* operation room, *COPD* chronic obstructive pulmonary disease, *SD* standard deviation

**Table 3:** The surgical procedure and outcomes

		mean ± SD (minimum, median, maximum)
		% (n/N)
Number of subjects	*N*	200
Intervention	% (n/N)	
Ileocecal resection		7.0% (14/200)
Right hemicolectomy		14.0% (28/200)
Sigmoid resection		21.5% (43/200)
High anterior resection		11.0% (22/200)
Low anterior resection		20.5% (41/200)
Other		26.0% (52/200)
Emergency surgery	% (n/N)	0.0% (0/200)
ASA-PS classification	% (n/N)	
Class 1		33.5% (67/200)
Class 2		61.0% (122/200)
Class 3		5.5% (11/200)
Class 4		0.0% (0/200)
Class 5		0.0% (0/200)
Length of small incision, cm	Mean ± SD	4.28 ± 1.16 (1.2, 4.00, 12.0)
Duration of surgery, minutes	Mean ± SD	194.0 ± 68.5 (91, 183.0, 560)
Blood loss, mL*1	Mean ± SD	43.65 ± 98.31 (0.0, 10.00, 944.0)
Blood transfusion, mL	Mean ± SD	0.2 ± 2.1 (0, 0.0, 30)
Drainage impl ant	% (n/N)	58.0% (116/200)
Conversion to open surgery	% (n/N)	0.5% (1/200)
Close of mesentery	% (n/N)	0.5% (1/200)
Colon exfoliation from splenic flexure	% (n/N)	16.0% (32/200)
Construction of stoma	% (n/N)	10.5% (21/200)
INTERCEED application	% (n/N)	
Under small incision on omentum		71.0% (142/200)
Under small incision on non-omentum		32.0% (64/200)
Intestinal anastomosis		0.0% (0/200)
Retroperitoneum		0.0% (0/200)
Exfoliation on pelvic floor		1.5% (3/200)
Exfoliation on lymph node dissection site		0.0% (0/200)
Exfoliation on other adhesions site		0.0% (0/200)
Other		1.0% (2/200)
INTERCEED removal due to stained black	% (n/N)	0.0% (0/200)
Previous adhesion	% (n/N)	
Application site		5.5% (11/200)
Non-application site		23.0% (46/200)
Additional INTERCEED application	% (n/N)	0.0% (0/200)

*ASA-PS* American Society of Anesthesiologists physical status, *SD* standard deviation

The incidence rate of postoperative adhesive intestinal obstruction was 1.0% (2/200) with a mean 6-month follow-up period. The total SSI rate was 3.5% (7/200), the deep incisional SSI rate was 0.0% (0/200), and the organ SSI rate was 0.0% (0/200). The incidence of anastomotic leak was 1.0% (2/200). Reoperation was performed in two cases: one for anastomotic leak and the other as cardiac surgery due to heart disease. The postoperative complications are shown in Table 4.

**Table 4:** The incidence rate of postoperative complications

	Mean ± SD (minimum, median, maximum)	
Number of subjects	200	
Follow-up period, days	182.8 ± 34.6 (5, 185, 355)	
AIO	1.0% (2/200)	0.12–3.57%
Revision surgery	1.0% (2/200)	0.12–3.57%
Related to AIO	0.0% (0/200)	0.00–1.83%
Related to postoperative bleeding	0.0% (0/200)	0.00–1.83%
Related to anastomotic leak/ drainage	0.5% (1/200)	0.01–2.75%
Other reason (cardiac surgery due to heart disease)	0.5% (1/200)	0.01–2.75%
SSI	3.5% (7/200)	1.42–7.08%
Superficial incisional SSI	3.5% (7/200)	1.42–7.08%
Deep incisional SSI	0.0% (0/200)	0.00–1.83%
Organ SSI	0.0% (0/200)	0.00–1.83%
Anastomotic leak	1.0% (2/200)	0.12–3.57%
Other Adverse events	7.0% (14/200)	3.88–11.47%
Aspiration pneumonia	1.0% (2/200)	0.12–3.57%
Paralytic ileus	1.0% (2/200)	0.12–3.57%
Urinary tract infection	1.0% (2/200)	0.12–3.57%
Anastomotic hemorrhage	0.5% (1/200)	0.01–2.75%
Infective endocarditis	0.5% (1/200)	0.01–2.75%
Prolapse of stoma	0.5% (1/200)	0.01–2.75%
Enteritis	0.5% (1/200)	0.01–2.75%
Clostridium difficile-associated diarrhea	0.5% (1/200)	0.01–2.75%
Acute heart failure	0.5% (1/200)	0.01–2.75%
Myocardial infarction	0.5% (1/200)	0.01–2.75%
Disorder of liver function	0.5% (1/200)	0.01–2.75%
All adverse events	12.0% (24/200)	7.84–17.33%
Death	1.5% (3/200)	0.31–4.32%
Aspiration pneumonia	0.5% (1/200)	0.01–2.75%
Cancer recurrence	0.5% (1/200)	0.01–2.75%
Sudden death	0.5% (1/200)	0.01–2.75%

*AIO* adhesive intestinal obstruction, *SSI* surgical site infection

## Discussion

The incidence of postoperative adhesive small intestinal obstruction after laparoscopic colorectal surgery has been reported to be 0.4–2.5% [22–26]. In two studies with a follow-up period of ≥ 2 years, the incidence of postoperative adhesive small intestinal obstruction was 2.4–2.5% [23, 24]. In this study, the incidence of postoperative adhesive small intestinal obstruction was 1.0% (2/200).

There is ample evidence that INTERCEED^®^ decreases the frequency of adhesion formation [4, 27]. This evidence has mainly been shown in gynecological surgery, but there are very limited data on its application in colorectal surgery. Three randomized controlled trials have explored the application of hyaluronate carboxymethylcellulose in colorectal surgery, finding that the frequency and extent of adhesion were significantly reduced in a meta-analysis [15, 16, 20]. The application of INTERCEED^®^ in laparoscopic colorectal surgery has only been described in the report of a single randomized controlled trial with a small number of cases, and the postoperative adhesive small intestinal obstruction rate was 0.0% (0/50) in the INTERCEED^®^ group versus 4.1% (2/49) in the control group [21]. The present study is the first report of a relatively large series using INTERCEED^®^ in colorectal surgery.

Regarding any serious adverse events, the Cochrane database of systematic reviews in barrier agents for adhesion prevention after gynecological surgery reported no adverse events directly attributed to adhesion agents [27]. Five trials studied the incidence of serious adverse events after the application of hyaluronate carboxymethylcellulose in colorectal surgery [4]. The difference between the hyaluronate carboxymethylcellulose group and the control group in the incidence of serious adverse events was non-significant. Naito et al. [21] reported that adverse events in the INTERCEED^®^ group of 50 cases included three cases (6.0%) of anastomotic leakage and 1 (2.0%) intra-abdominal abscess. In the present study, anastomotic leakage occurred in two cases. There were no cases of intra-abdominal abscess formation or any serious adverse events that could be directly attributed to INTERCEED^®^. This shows that INTERCEED^®^ can be safely used in colorectal surgery.

Hyaluronate carboxymethylcellulose, which is mainly used in gastrointestinal surgery, has been used in laparotomy, but it was found to be unsuitable for manipulation through trocars, as is required in laparoscopic surgery [21]. In contrast, INTERCEED^®^ is a woven sheet of oxidized regenerated cellulose that lends itself well to use in laparoscopic surgery because its softness and pliability facilitate intraoperative manipulation [21]. However, there is considered to be no significant difference between hyaluronate carboxymethylcellulose and INTERCEED^®^ in terms of intraoperative manipulation when inserting via an umbilical small incision.

The main limitations of the present study were that it was not randomized and that it lacked a prospective case-matched control group, thus failing to provide definitive proof of any benefit. As such, further multi-institution, randomized studies are needed to confirm whether or not INTERCEED^®^ can indeed reduce the postoperative adhesive small intestinal obstruction rate in laparoscopic colorectal surgery. However, the authors believe that the findings of the present study will provide a firm foundation for future studies.

## Conclusion

Using INTERCEED^®^ in laparoscopic colorectal surgery is safe and may be useful for preventing postoperative adhesive small intestinal obstruction. A randomized controlled study is needed to further evaluate the true clinical significance of using INTERCEED^®^ in laparoscopic colorectal surgery.
